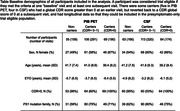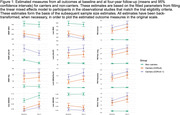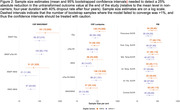# Sample Size Estimates for Detecting Removal of Pathology in Clinical Trials of Autosomal Dominant Alzheimer’s Disease

**DOI:** 10.1002/alz.095101

**Published:** 2025-01-09

**Authors:** David M Cash, Katy E Morgan, Thomas Veale, Ian B. Malone, Tammie L.S. Benzinger, Laura Ibanez, Yan Li, Guoqiao Wang, Eric McDade, Randall J. Bateman, Chris Frost, Nick C Fox, Dominantly Inherited Alzheimer Network

**Affiliations:** ^1^ Dementia Research Centre, UCL Queen Square Institute of Neurology, London United Kingdom; ^2^ UK Dementia Research Institute at UCL, London United Kingdom; ^3^ London School of Hygiene and Tropical Medicine, London United Kingdom; ^4^ Mallinckrodt Institute of Radiology, Washington University in St. Louis, St. Louis, MO USA; ^5^ Washington University in St. Louis, School of Medicine, St. Louis, MO USA; ^6^ Knight Alzheimer Disease Research Center, St. Louis, MO USA; ^7^ Washington University in St. Louis School of Medicine, St. Louis, MO USA; ^8^ UK Dementia Research Institute, Queen Square Institute of Neurology, University College London, London United Kingdom

## Abstract

**Background:**

Effective treatments are now available, which have demonstrated reductions in amyloid plaque burden while slowing cognitive decline in early symptomatic Alzheimer’s disease (AD). Intervening before onset of cognitive impairment could provide greater benefit, particularly for individuals who carry an autosomal dominant mutation known to cause AD. To better guide the design of upcoming prevention trials, reliable sample size estimates for detecting relevant reductions in pathology are needed.

**Method:**

Longitudinal PIB PET and CSF biomarker data were obtained from the Dominantly Inherited Alzheimer Network Observation study (datafreeze 14, see Table). Participants were included in the analysis based on eligibility criteria from DIAN‐TU‐001: estimated years to expected onset (EYO) between ‐15 to +10 and global Clinical Dementia Rating (CDR) score between 0 and 1, inclusive. Sample size estimates were also obtained for trials with individuals having CDR = 0 only. Linear mixed‐effects models were used to estimate baseline values and rates of change in outcome measures for mutation carriers and non‐carriers. Outcomes included CSF biomarkers of amyloid and p‐tau 181 using three assays (INNOTEST, XMAP and Lumipulse) and Standardized Uptake Value Ratio (SUVR, cerebellar grey matter reference region) of PIB PET from six regions. We then used these estimates to compute sample size estimates to detect a 25% reduction in pathology by four years, assuming 5% significance, 80% power, and 40% dropout. Uncertainty in sample size estimates was quantified through bootstrapping.

**Result:**

There were large differences between carriers and non‐carriers at baseline and the end of a four‐year study (Figure 1). Sample size estimates were consistently higher in scenarios involving only CDR = 0 carriers (Figure 2). For PIB PET cortical mean, 40[95%CI: 26,66] pariticpants per arm would be needed to detect a 25% reduction and (63[40,115] for the CDR = 0 subsample. Similar estimates were observed for individual brain regions. XMAP Aβ42 (CDR 0‐1: 21, [11,65], CDR 0: 51 [21,333]) and Lumipulse Aβ42‐40 ratio (CDR 0‐1: 22 [13,46], CDR 0: 47[25,104]) were the most promising CSF outcome measures.

**Conclusion:**

Sample size estimates needed to detect a 25% reduction in pathology levels in prevention studies are in the range of 30‐40 participants per arm.